# Multidimensional evaluation of performance: experimental application of the balanced scorecard in Ferrara university hospital

**DOI:** 10.1186/1478-7547-7-15

**Published:** 2009-09-08

**Authors:** Adriano Verzola, Roberto Bentivegna, Gianni Carandina, Lucio Trevisani, Pasquale Gregorio, Alberto Mandini

**Affiliations:** 1 Management Planning and Control, St Anna University Hospital, Ferrara, Italy; 2 Medical Direction Committee, St Anna University Hospital, Ferrara, Italy; 3 Analysis Laboratory, St Anna University Hospital, Ferrara, Italy; 4 Digestive Endoscopy, St Anna University Hospital, Ferrara, Italy; 5 Post Graduate School in Hygiene and Preventive Medicine, Ferrara University, Ferrara, Italy

## Abstract

**Background and Aims:**

One of the best-known performance planning and evaluation techniques utilising both monetary and non-monetary data is the *Balanced Scorecard (BSC)*. This is a means of rationalising the global activity of a business in the attempt to create value, and to translate the company vision into a set of tactical objectives and measurable strategies. The aim of this study was to implement and evaluate the use of BSC in two departments of the St. Anna University Hospital, Ferrara: the Analysis Laboratory and Digestive Endoscopy operating units (OU).

**Materials and methods:**

With the collaboration of the health workers involved, a precise methodological programme was pursued: Definition of the strategic map from 4 perspectives, according to Kaplan and Norton, Definition of the Key Performance Areas (KPA), or macro-objectives, Identification of the cause-effect relationships between KPAs, Identification of the sub-objectives of each KPA, Definition of the Key Performance Indicators (KPI), Definition of the weight/importance of each objective in the global evaluation.

**Results:**

The information gathered permitted the definition of macro- and sub-objectives for each perspective, as well as determining the relevant indicators, standards, weights, frequency of detection and means of acquisition. Strategic maps showing the cause/effect relationships in each OU were created, as were 'evaluation panels', which describe the global performance of each department. For each perspective, the fundamental data were summarised in one table. Evaluation of each perspective yielded a positive result for the majority of the objectives, and the global result (including all 4 perspectives) was found to be satisfactory.

**Discussion-Conclusion:**

The Balanced Scorecard was implemented in the abovementioned OUs of St. Anna University Hospital, Ferrara, after the health workers themselves realised the need for change.

In our research the employees were pleased to be evaluated, not only for the financial outcomes, but also for the satisfaction of improving internal procedure, relationships with the community and their own growth/learning. BSC is an ideal point of contact between the financial and clinical dimensions of management. However, difficulties in its application were faced, among these, at least in the initial phase, the lack of information systems able to drive it, and the complexity of the research for specific indicators needed to be overcome. The time factor (on average, at least two years are required) and the availability of technological resources were also limiting factors.

The rapid diffusion of BSC among the principal international profit and non-profit organisations is testament to its great potential. This project could be seen as a preparatory phase in the strategical analysis of a subsequent business plan.

## Background and Aims

In order to overcome the limitations of financial information, many organisations plan their activities and evaluate their performance by integrating their budget and accounting information with other performance indicators pertaining to phenomena or activities able to influence future performance.

One of the best-known performance planning and evaluation techniques utilising both monetary and non-monetary data is the *Balanced Scorecard (BSC)*. This is a means of rationalising the global activity of a business in the attempt to create value, translating the company vision into a set of tactical objectives and measurable strategies.

Using the Balanced Scorecard permits evaluation of the performance of an organisational structure, both from a strategic and from an operative perspective. In fact, it reflects the equilibrium reached between short and long-term objectives via financial and non-financial measures, via indicators of delay, trend, and internal and external performance [1].

A fundamental element of a BSC system is the creation of a strategic map, a document which presents an overview of the objectives which the firm intends to pursue, in each perspective, in order to create a business strategy [2].

The strategic map permits the following perspectives to be interlinked:

• The financial perspective;

• The client/community perspective;

• The internal procedure perspective;

• The growth and staff learning perspective.

Our firm heeded the growing request of its workers to implement a course of action to facilitate the practical and operative impact of the theoretical know-how regarding this tool.

In virtue of the characteristics of this pilot study, development of the strategic map was limited to two internal structures, or Operating Units (OU): the Centre for Digestive Endoscopy and the Analysis Laboratory.

The aim of this study was to implement a new performance management tool, the BSC, in the St. Anna University Hospital, Ferrara.

This was necessitated by the existence of the following situations in need of improvement:

• The health service as a multi-dimensional product;

• The internal directional control mechanisms;

• A management system linked to financial measures which furnishes information about accomplished tasks.

## Materials and methods

Currently, in Ferrara University Hospital there is not a system of balanced measurement of performance. Numerous objectives, to be reached within the year, are defined for every department. Usually the objectives are classified in two types: economic and organizational and they are arranged between the direction and the department. A specific indicator for each objective has been defined. The global performance of Operating Unit (UO) has never been measured. The economic objectives have a great weight in comparison to the organizational objectives.

Taking the context and the inescapable constraints (regulation, indications, organisation and resources) into account, a series of objectives and tasks was defined via multi-dimensional analysis.

A monitoring system comprising specific, previously identified indicators (the panel) consented awareness and modulation of the performance, progress and implementation of the plan [3].

The structure of the strategic map and the importance submitted to the analyzed perspectives, are the principal differences between the managerial model born in Private Firms and the public hospitals system. The division into the four classical perspectives remains, but a different vision of the priorities must be produced. If in a private firm the financial economic perspective has a primary role, in health care service the patient and the community have primary importance. The satisfaction of necessities and requests is the first objective.

With the collaboration of the health workers, a precise methodological procedure was pursued:

• Definition of the strategic map, divided into 4 perspectives according to Kaplan and Norton [4]:

• The community perspective: it defines the value which the health care service proposes to the community. The relationship between community and sanitary organization show numerous facets. The public health service must find all these relationships.

The community perspective was further subdivided into:

• Clients as Users: it represents the individual and direct relationship between the Clients and the organization.

• Clients as Owners: Clients are the "Owners" of the public service. They can verify the results of strategies, policy and quality assurance, favouring the continuous improvement of resources allocation.

• Clients as Public entities: The application of the norms and the laws protects the community and the environment to the purpose to improve the relationship with the performance of sanitary organization.

The objectives defined in these three dimensions improve the description of the meaning of the community perspective

• The internal procedure perspective: Through the internal procedure perspective the specific procedures are individualized and performed for Clients as Users, Clients as Owners and Clients as Public entities. An objective of the internal procedure perspective can have a direct influence on the community perspective.

• The financial perspective: In Public Healt Service, the financial resources represent some ties inside which the organization must operate. It is a primary tool of the efficiency. The *Budget result *is the first objective of the financial perspective

• The growth and learning perspective: The growth and learning perspective describes the "intangible wealth" of the sanitary organization.

The growth and learning perspective was divided into:

• Human resources: all the competences and the necessary abilities to correctly perform the strategy

• Information resources: all the procedures and the computer infrastructure to support the necessary informative flows to take effective decisions

• Organisational resources: the business culture, the leadership, the ability to work in team, the alignment of the organization with the strategy.

In our study, the growth and learning perspective needs further specific researches to define other objectives and indicators (in particular the Information resources). During 2007 a specific indicator for the continuous improvement of ICT, especially in the relationships with GPs, has been proposed. This study will be completed during the 2008.

In the case of the Analysis Laboratory Operating Unit (OU), "user" did not only refer to the patient, but also to the other entities availing themselves of the services of the OU on a daily basis, such as GPs and hospital physicians.

• Definition of the Key Performance Areas (KPA) or macro-objectives, i.e. the most important fields in which to measure the performance levels, linking them to the perspectives identified [5].

• Determination of the cause/effect relationships between the KPAs: in this way it's possible to understand the connection between the realization of the objectives of a performance area and the realization of another area. This factor shows the interdependence inside our public hospitals system.

• Indication, for each KPA, of the pre-established sub-objectives required by the OU to fulfil the KPAs.

• Definition of the Key Performance Indicators (KPI) to monitor the degree of achievement of the performance levels defined for each KPA.

Based on the above information and criteria, indicators, and consequently specific objectives, were produced, classified into common areas such as measurability, reliability, practicability, sustainability and relevance.

The data were organised in a spreadsheet as follows:

○ 1^st ^column: macro-objective defined in the strategic map;

○ 2^nd ^column: pre-established sub-objectives - mainly objectives from areas in which the services were already dealing with: budget, credit, performance indicators;

○ 3^rd ^column: definition of the indicator for evaluating the achievement of the objective (clarification of the previous point);

○ 4^th ^column: value found;

○ 5^th ^column: standard/expected value. The standard value represented the acceptable threshold, i.e. that which defined the upper and lower-most limits of adequate quality of assistance. In this study, the standard was established in agreement with the health workers, either by analysing reported data or based on the previous experience of the OU. In the case of new measures which cannot not be based on previous experience or reports, in future it could be necessary to readjust the fixed standard to bring it in line with operative reality;

○ 6^th ^column: weight/importance to attribute to each objective; the sum of weights for each perspective was equal to 100. In order to define the weight it was decided to consider the mean weight for each indicator supplied by a pool of professionals (opportunely involved) from various sectors: assistance, organisational, directional. The attribution of a weight to each objective permitted balanced evaluation of OU activity, highlighting the key objectives of the relevant perspective;

○ 7^th ^column: means of detection. The data were revealed either by information systems, directly from the employee or from specific informative sources (Quality Assurance Office, Medical Direction, Mixed Advisory Committee, Public Relations Office and/or Management Control);

○ 8^th ^column: frequency of data acquisition.

In order to evaluate the results for each perspective, the fundamental data were grouped and summarised in one table. The evaluation panel was made up of 5 of these tables, one for each perspective and one summarising the progress of the OU as a whole.

The achievement of objectives was graphically represented as a traffic light as follows:

○ **Red**: objective not achieved

○ **Green**: objective achieved

○ **Amber**: "borderline" conditions:

• The observed value was slightly out of line with the established target

• Unsurmountable difficulties impeded achievement of the objective.

The data for the Digestive Endoscopy OU were obtained in 2007.

Those for the Analysis Laboratory OU were partly obtained in 2007, and partly in the first six months of 2008: in fact, some indicators refer to activities not implemented prior to 2008.

In order to arrive at an overall performance value for each perspective, the weights of the various objectives of each perspective were added and translated into a traffic light, the colour of which represents the prevalent colour obtained. These indications of global achievement are presented in the summary table of each OU.

### Centre for Digestive Endoscopy

Additional file 1 shows the strategic map elaborated for the Digestive Endoscopy Unit, highlighting the macro-objectives and cause-effect relationships.

Additional files 2, 3, 4, 5 show and describe the specific objectives, the indicators, the observed values, the standard, and the weight for each perspective.

### Analysis Laboratory

Additional file 6 shows the strategic map elaborated for the Analysis Laboratory highlighting the *macro-objectives *and the *cause-effect relationships*.

Additional files 7, 8, 9, 10 show and describe the specific objectives, the indicators, the observed values, the standard, and the weight for each perspective.

Additional files 11 and 12 show the *EVALUATION PANEL*. containing the *level of performance *of each perspective of the Centre for Digestive Endoscopy and the Analysis Laboratory respectively.

## Results

### Centre for Digestive Endoscopy

*Community perspective *(Additional file 2): as can be seen from the panel, 4 objectives were achieved and their traffic lights were therefore coloured green. The other two objectives, in contrast, were not achieved as expected: for this reason, their traffic lights were coloured red.

On the whole, the level of performance from the community perspective was found to be fully satisfactory, and was therefore indicated by a green traffic light: this overall evaluation was linked to the fact that the sum of the weights of the objectives achieved and indicated in green (sum: 70) was far higher than that of the objectives not achieved (30) (Additional file 11).

*Internal procedure perspective *(Additional file 3): ten objectives were achieved (sum of weights: 68), three showed border-line values (sum: 19) and one required further improvement (weight: 13). On the whole, the performance of this perspective was considered fully satisfactory and was thus assigned a green traffic light (Additional file 11).

*Financial resource perspective *(Additional file 4): two objectives showed a value midway between alignment and misalignment with the standard (amber traffic light, sum: 72). One objective, however, was not achieved (weight 28). In defence of the Endoscopy Unit, it should be noted that the lack of achievement of the objective "Increase 2006 production levels" was linked to understaffing problems during the year. This perspective was characterised, as whole, by an amber traffic light (Additional file 11).

*Growth and Learning Perspective *(Additional file 5): all three objectives were fully achieved. The performance from this perspective was therefore considered good and indicated with the colour green (Additional file 11).

### Analysis Laboratory

*Community perspective *(Additional file 7): as can be seen from the panel, 6 objectives were achieved and their traffic lights were therefore coloured green (sum: 52). One objective, however, revealed a condition of consistent misalignment with the expected target: for this reason, the traffic light was coloured red (value: 12). The other 3 objectives showed a borderline condition (amber, sum: 36). As a whole, the level of performance of this perspective was assigned an amber traffic light, highlighting a borderline situation in need of close monitoring (Additional file 12).

*Internal procedures perspective *(Additional file 8): the vast majority of the objectives were achieved and thus assigned the colour green (sum: 86). As a whole, the level of performance of the internal procedures perspective was considered fully satisfactory and thus assigned a green light (Additional file 12).

*Financial resources perspective *(Additional file 9): three objectives showed values in line with the standard (green traffic light, sum: 55), two, however, were characterised by an intermediate value between alignment and misalignment with respect to the standard (amber traffic light, sum: 45). As a whole, the level of performance was assigned a green light (Additional file 12).

*Growth and Learning Perspective *(Additional file 10): five objectives were achieved (green traffic light, sum: 80), one had an intermediate value between alignment and misalignment with respect to the standard. As a whole the performance level was considered satisfactory (green traffic light - Additional file 12).

## Discussion

Analysis of the impact of the BSC on the pilot OUs was carried out along three parallel lines:

- Based on the results obtained from experience, which consented quantification and analysis of the global performance of the service

- Comparison with the opinions of the clinician involved in implementation of the project.

- The relationship with the needs for development and the prospectives of estimated improvement for the organisation.

Bearing the above information in mind, it was possible to describe the strengths and weaknesses of the applied experience objectively.

### Strengths

In order to enable collaboration between the various organisational figures, it was necessary to form a business strategy which was accessible to the health workers involved [6].

The BSC offers this opportunity: it brings the business strategy to the objectives attributed to each operative unit, becoming a tool orientated to guide managerial action. Thanks to the BSC, the strategy may be communicated to all levels of the organisation, and allocation of resources can reflect the strategic aims [7].

The experience of the Balanced Scorecard, in St. Anna University Hospital, Ferrara, was provoked by the request for change by the health workers involved.

The first motivation was the necessity of communicating the strategy to all the levels of the Operative Unit. There was a need to involve all health workers in improving business performance, and to fulfil this objective, indicators and data connected to daily activity were required.

The BSC, via the strategic map and the sequence of cause-effect relationships, permitted explanation to all levels of the organisation that, for example, improving the relationship with the users increases the patients' trust [7]. The strategic map connects all the objectives; in the same way, every action leads to reaction in daily clinical practice.

On a deeper analytical level, the simple provision of information either about the Operative Unit upon admission or about the diagnostic exam to be carried out can lead to far greater satisfaction for the patient, and an increase in the attractivity of the structure, which may thus lead to an improvement in the financial performance.

The BSC permits greater facility in explicating the cause-effect relationships and centres on the clinician, staff and the patient [3].

The main limitation of traditional management models, linked mainly to negotiation of the budget, is their lack of clarity for the health worker. Evaluating the activity of endoscopy via, for example, the percentage of times the caecum is reached, facilitates the consensus of the operative, who is more likely to understand this type of challenge than one relating to financial data. This could be the start of an innovative phase in the relationship between the healthcare workers and their profession, characterised by the maturity of spontaneously seeking or identifying systems of measurement of their performance.

The originality of BSC is linked to its multi-dimensionality, i.e. its ability to represent the performance of an organisation from various points of view, as well as its capacity to offer a strategic vision of performance, explaining business strategy, after its formulation, and finding an efficacious means of putting it into practice [6].

The multi-dimensionality of BSC was particularly helpful in finding a common language for the different professional figures working within our organisation (doctors, nurses, economists, etc.), which made it possible to reach agreement on strategic priorities and the means of their execution [8].

The desire was to fuse the result of the cultural (refresher courses, guidelines etc), interpersonal (rapport with patients or other agencies), organisational (understanding the language and practicality of the organisational pathways), managerial (perception of the structure in question in the general healthcare and economic context of the country) improvement in a harmonious fashion.

The BSC may represent an ideal point of contact between the financial and clinical dimensions of management [9].

### Weaknesses

This new management tool presents the following risks:

It could be interpreted by health workers as a system of measurement proposed by the management and thus as a potential work overload. This must be avoided by explaining the model to all levels of personnel, highlighting the improvement it can bring to the organisation.

Interiorisation of the aims and logic behind the Balanced Scorecard and consensus behind its application are two fundamental criteria for the success of the project.

Another difficulty was, in this case, linked to the absence, at least in the initial phase, of information systems able to drive the BSC effectively. This shortcoming necessitated laborious extrapolation of data.

One criticality that could be met by the health workers when using such a tool is linked to the difficulty in finding specific indicators and/or their related values as a direct consequence of the aforementioned lack of information. The scarce availability of information regarding some fields may hinder, or even impede, implementation of the project. These conditions may occur, for example, in the absence of filing systems (databases), or in cases where information is stored solely in hard copy.

One factor which could potentially limit the reliability of results of this methodology may be represented by the objective difficulty in the choice or definition of reference standards, in particular when they are not available in literature or law, or from institutional objectives or Benchmarking investigations. In these cases the research is developed, when possible, from historical analysis or from professional experience.

Possible criticalities may also emerge in the definition of weights attributed to each objective, as the factors involved are subjective.

The time factor (on average, at least two years are required) and the availability of human and technological resources constitute severe limiting factors which may lead management to other planning solutions.

### Opportunity

Introduction of BSC promotes the processes of organisational and managerial integration between separate bodies of an organisation, thanks to the development of communicative interfaces; in the same way it increases internal communication within these bodies, encouraging progressive improvement in quality of efficiency and efficacy.

BSC transmits, to both workers and managers alike, the possibility of enriching the perception of external requirements: it consents harmonious integration between the performance quality perceived by the users of the service and the organisational system within the service itself.

The results obtained from application of this model also highlighted an opportunity for the health workers and management to increase attention to "intangible assets" such as, for example, research and training. The possibility of quantifying these assets permitted, consequently, promotion of growth, improving the intellectual resources of the organisation and its measurement using an agreed scale of reference.

### Threats

Potential negative effects of BSC introduction could arise during the process of "interiorisation" of the model due to the non-uniformity of the organisation as a whole. This would lead to an unbalanced development of the improvement process of the various services offered and a condition of progressive "isolation" of some of these. Another risk could stem from managerial decisions resulting from the Operative Unit pilot scheme initiative: the time and resources required for detailed analysis of the comparison between strengths and weaknesses, and between advantages and disadvantages of the entire organisation could lead to its non-implementation on a global scale.

## Conclusion

In our study, the health workers were pleased to be evaluated, not only for the financial rewards, but also for the opportunity to improve internal procedure, relationships with the community and growth/learning. This approach was perceived as increased attention of the management on intangible assets, with the knowledge that the value of a health care service resides in its personnel, in their relationship with each other and with the users of the service. Application of the BSC could lead to distribution of financial incentives taking into account all four of the perspectives in a balanced fashion.

The rapid diffusion of BSC in the main global profit and non-profit corporations is testament to its great potential.

Based on the results obtained, this project could be seen as a preparatory step in a strategic analysis of the organisation as a whole.

## Abbreviations

BSC: Balanced Scorecard; OU: Operating Units; KPA: Key Performance Areas; KPI: Performance Indicators; MAC: Mixed Advisory Committee; PRO: Public Relations Office; CME: Continuing Medical Education; TAT: Internal Turn-Around Time; CV: Coefficient of Variation.

## Competing interests

The authors declare that they have no competing interests.

## Authors' contributions

AV, RB, AM drafted the manuscript. LT, GC, RB, AV and AM conceived of the study, performed its design and the coordination, acquired, analyzed and interpreted the data. PG conceived of the study, and participated in its design. All authors read and approved the final manuscript.

## Supplementary Material

Additional file 1 **Strategic map of digestive endoscopy OU**. the file represents the final outcome of strategic map of digestive endoscopy. Click here for file

Additional file 2 **Digestive endoscopy OU - community perspective**. the file represents all indicators researched and results obtaines regarding objectives of community perspective of digestive endoscopy. Click here for file

Additional file 3 **Digestive endoscopy OU - internal procedure perspective**. the file represents all indicators researched and results obtaines regarding objectives of internal procedure perspective of digestive endoscopy. Click here for file

Additional file 4 **Digestive endoscopy OU - financial resource perspective**. the file represents all indicators researched and results obtaines regarding objectives of financial resource perspective of digestive endoscopy. Click here for file

Additional file 5 **Digestive endoscopy OU - growth and learning perspective**. the file represents all indicators researched and results obtaines regarding objectives of growth and learning perspective of digestive endoscopy. Click here for file

Additional file 6 **Strategic map of analysis laboratory OU**. the file represents the final outcome of strategic map of analysis laboratory. Click here for file

Additional file 7 **Analysis laboratory OU - community perspective**. the file represents all indicators researched and results obtaines regarding objectives of community perspective of analysis laboratory. Click here for file

Additional file 8 **Analysis laboratory OU internal procedure perspective**. the file represents all indicators researched and results obtaines regarding objectives of internal procedure perspective of analysis laboratory. Click here for file

Additional file 9 **Analysis laboratory OU -financial resource perspective**. the file represents all indicators researched and results obtaines regarding objectives of financial resource perspective of analysis laboratory. Click here for file

Additional file 10 **Analysis laboratory OU - growth and learning perspective**. the file represents all indicators researched and results obtaines regarding objectives of growth and learning perspective of analysis laboratory. Click here for file

Additional file 11 **Digestive endoscopy OU - BSC**. the file represents the global performance reached in four perspectives of strategic map of digestive endoscopy. Click here for file

Additional file 12 **Analysis laboratory OU - BSC**. the file represents the global performance reached in four perspectives of strategic map of analysis laboratory. Click here for file
