# Supplementary figures and images for: Multidimensional evaluation of performance: experimental application of the balanced scorecard in Ferrara university hospital
Source: Cost Eff Resour Alloc. 2009 Sep 8;7:15. doi: 10.1186/1478-7547-7-15 (PMC2759901; doi:10.1186/1478-7547-7-15)

COMMUNITY PERSPECTIVE

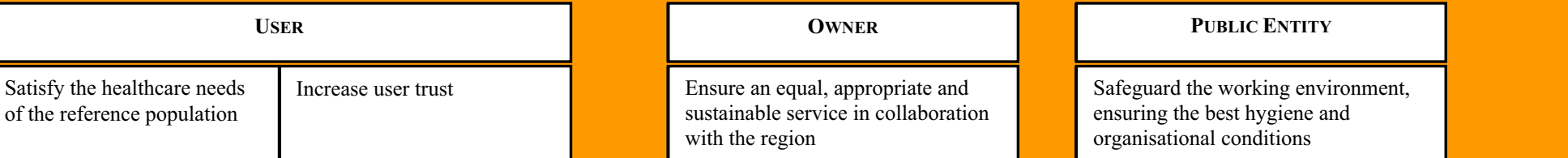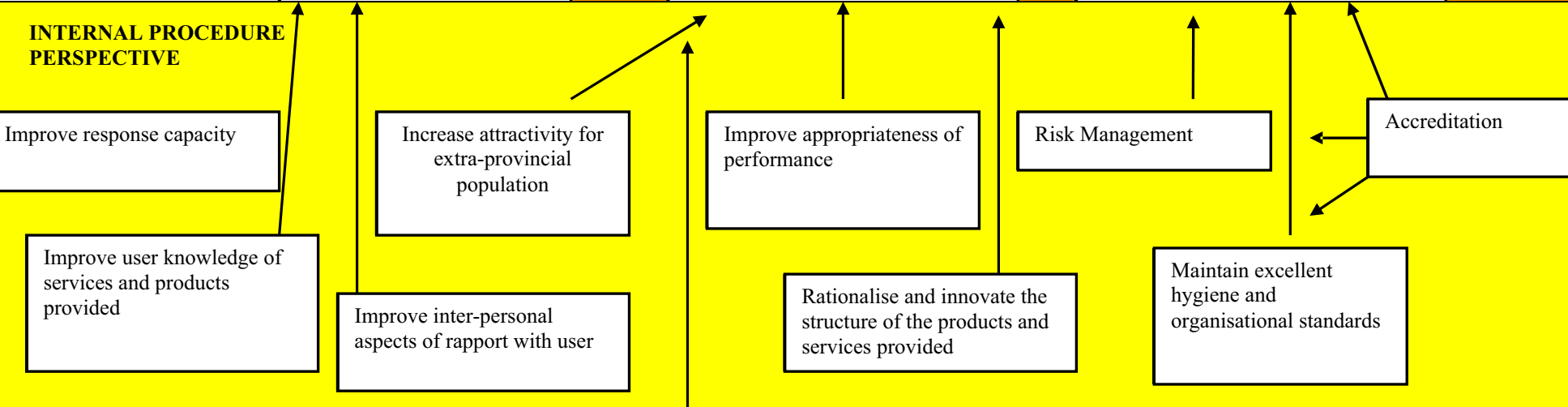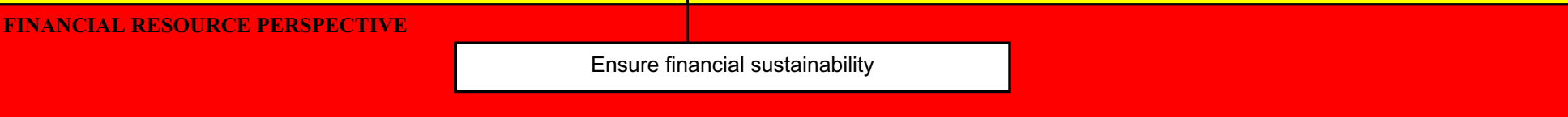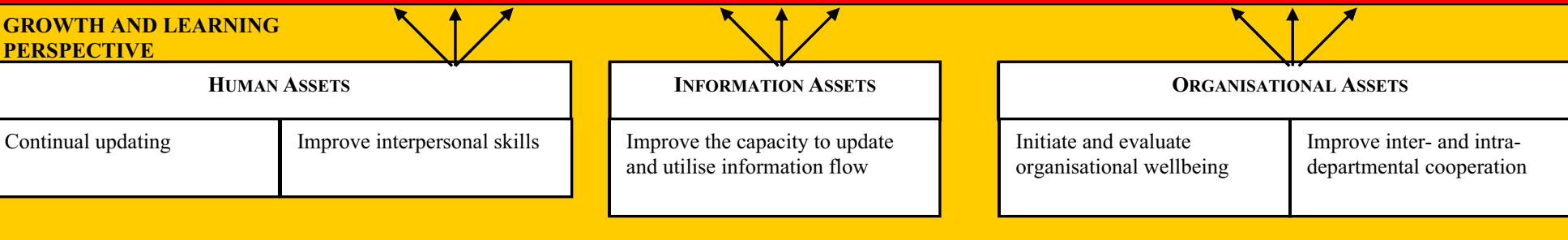

Supplement: Additional file 1 — Strategic map of digestive endoscopy OU. the file represents the final outcome of strategic map of digestive endoscopy. [file 1478-7547-7-15-S1.pdf]
