# Supplementary material for: Multidimensional evaluation of performance: experimental application of the balanced scorecard in Ferrara university hospital
Source: Cost Eff Resour Alloc. 2009 Sep 8;7:15. doi: 10.1186/1478-7547-7-15 (PMC2759901; doi:10.1186/1478-7547-7-15)
Supplement: Additional file 2 — Digestive endoscopy OU - community perspective. the file represents all indicators researched and results obtaines regarding objectives of community perspective of digestive endoscopy. [file 1478-7547-7-15-S2.pdf]

| Macro-Objective | Specific Objective | Indicator | Observed value | Standard | Weight | Pictorial representation |
|---|---|---|---|---|---|---|
| **USER**<br><br>Satisfy the healthcare needs of the reference population<br><br>Increase user trust | Clear and timely information on the wards | Number of patients who received information sheets/number of patients | 100% | 95% of patients | 20 | 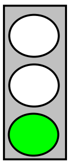 |
| | Overall client satisfaction | Satisfaction expressed by the users on instrument cleanliness | 1.34 | <2 | 30 | 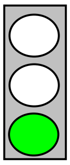 |
| | Increase user trust | N° of complaints | 2007: 3 complaints | Zero complaints/year | 22 | 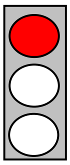 |
| **OWNER**<br><br>Ensure an equal, appropriate and sustainable service in collaboration with the region | Budget result | Comparison of final and estimated balances | 4.13% difference | alignment | 8 | 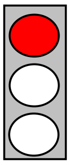 |
| | Transparency | Employees in contact with public who wear ID badges/total employees | 100% | 95% of employees | 10 | 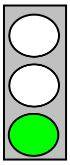 |
| **PUBLIC ENTITY**<br><br>Safeguard the working environment, ensuring the best hygiene and organisational conditions | Accessibility of hospital facility | Number of wheelchair-friendly lifts labelled "for public use" and bearing key locations/total number of lifts available for public use | 100% | 95% of lifts available for public use | 10 | 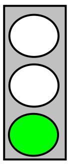 |

*COMMUNITY PERSPECTIVE*

The specific objectives selected in this area were:

1. *Clear and timely information on the wards:* for this objective the indicator chosen was the number of patients who had received information sheets (containing, for example, staff names, consulting times, information about procedure, regulations and patients’ rights) out of the total number of patients. The standard was 95% of patients, decided by the Mixed Advisory Committee (MAC) and the Public Relations and Quality Assurance

Offices, the weight attributed was 20%, the means of detection was a verification carried out by the MAC, and the frequency of acquisition was annual.

2. *Overall client satisfaction:* the means of detection of this objective was a Client Satisfaction Questionnaire administrated by the Quality Assurance Office. The users were asked to express their satisfaction (values from 1: very satisfied to 5: very unsatisfied), and the indicator chosen was satisfaction with instrument cleanliness, which was the criteria considered most important by the user. The standard was <2, the weight 30%, and the frequency of acquisition was annual.

3. *Transparency:* the indicator chosen was the number of staff members in contact with the public who wear ID badges out of the total number of staff members, the standard was 95% of staff members (defined by the MAC), the weight was 10%, the means of detection was verification carried out by the MAC, and the frequency of acquisition was annual.

4. *Accessibility of hospital facility:* the indicator chosen was the number of wheelchair-friendly lifts marked "for public use" and bearing the location of the keys out of total number of lifts available to the user, the standard was 95% of public lifts (defined by the MAC), the weight attributed was 10%, the means of detection was a systematic verification carried out by the Quality Assurance Office and the MAC, and the frequency of acquisition was annual.

5. *Budget result:* the indicator chosen was given by comparing the final balance with the estimated budget, the standard was 100%, the weight given was 8%, the means of detection was a verification carried out by Management Planning and Control, and the frequency of acquisition was annual.

6. *To increase user trust:* the indicator chosen was the number of complaints, the standard was “no complaints” (compared with previous years’ data, 2005 and 2006: no complaints), the weight attributed was 22%, the means of detection was a verification carried out by the Public Relations Office (PRO), and the frequency of acquisition was continual.
