# Supplementary material for: Multidimensional evaluation of performance: experimental application of the balanced scorecard in Ferrara university hospital
Source: Cost Eff Resour Alloc. 2009 Sep 8;7:15. doi: 10.1186/1478-7547-7-15 (PMC2759901; doi:10.1186/1478-7547-7-15)
Supplement: Additional file 3 — Digestive endoscopy OU - internal procedure perspective. the file represents all indicators researched and results obtaines regarding objectives of internal procedure perspective of digestive endoscopy. [file 1478-7547-7-15-S3.pdf]

Macro-Objective	Specific Objective	Indicator	Observed value	Standard	Weight	Pictorial representation
Rationalise and innovate the structure of the products and services provided<br><br>Increase attractivity for extra-provincial population	Increase n° of colonscopies carried out in sedated patients	Colonscopies in sedated patients in 2007/colonscopies in sedated patients in 2006	2006:564<br>2007:795<br>**+29%**	>5%	7	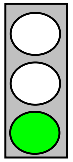
	Draft form for evaluation/verification of intestinal hygiene in admitted patients awaiting colonoscopy	Comparison of 2007 vs. 2006	The Draft form has been produced	Evidence: evaluation/verification of intestinal hygiene form	4	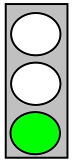
	Quality of procedure	% of cases where caecum is reached (except where unnecessary/impossibile) data: 2007	78.1%	>90%	13	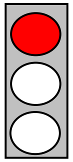
	Improve professional quality: maintain the system of clinical performance indicators (number of outpatient procedures cancelled)	Number of procedures cancelled/total number of procedures – data in on-line reports	2007: 7.13%	0%	4	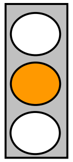
	Continue colon carcinoma screening programme	Results of questionnaire	The screening programme continues	Evidence: continuation of screening programme	5	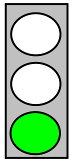
	Improve PEG patient management, reducing frequency of long-term complications	Increased n° of in- and outpatient "endoscopic consultations" recorded vs. 2006	2007: 122;<br>2006: 107;<br>+14%	>5%	9	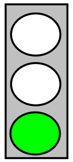

Accreditation	Revise chapters 1, 3 and 6 of Accreditation Manual	Internal inspection	The accreditation chapters have been revised	Evidence: revised chapters	10	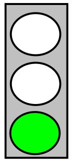
Improve inter-personal aspects of rapport with user<br><br>Improve user knowledge of services and products provided	Maintain levels of communication	Verification by MAC of information sheet provision upon patient admission	MAC report has been produced	MAC report	3	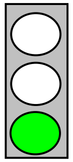
Improve appropriateness of performance	Investigation of unforeseen hospitalisations following endoscopy	Number of unforeseen hospitalisations following endoscopy /number of endoscopy patients	4 cases/5217 endoscopies (0.07%)	<0.45% (mean Project IQIP)	9	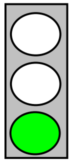
	Keep register of endoscopy-related complications	Internal register of Centre for Digestive Endoscopy	The Register has been produced	Evidence: Register of Complications Following Endoscopy	7	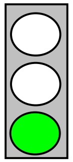

Improve response capacity	Optimal efficiency levels	Number of procedures carried out per operator/year	2 operators out of 9 did not reach the standard	100 gastroscopies/year 100 colonscopies/ year	6	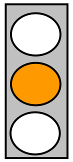
	Adherence to UAC waiting times (only for gastroscopies)	non-urgent gastroscopies with waiting times of < 60 days (outpatients)/non-urgent gastroscopies	100%	100%	13	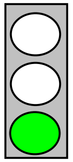
Risk Management	Reporting Sentinel Events	Specific reports of OU Manager on actions carried out	The specific reports have been produced	Evidence: specific reports	6	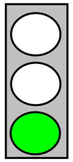
Maintain excellent hygiene and organisational standards	Risk management: endoscope decontamination	Number of colonies developed after decontamination	0%	0% colony development	4	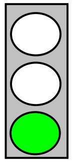

INTERNAL PROCEDURE PERSPECTIVE

The specific objectives identified were:

1. *To increase the number of colonoscopies carried out in sedated patients:* the indicator chosen was the number of colonoscopies carried out in sedated patients in 2007 with respect to the number of colonoscopies carried out in sedated patients in 2006. In recent years, due to progress in the pharmacological field and improvements in

the possibility of monitoring during colonoscopic investigation, in addition to patients being better informed, sedation has become a much more widely used practice [1]. The standard was >5% (agreed in the 2007 budget), the weight was 7%, the means of detection was a verification carried out by the Centre of Digestive Endoscopy, and the frequency of acquisition was annual.

2. *To draft a form to evaluate/verify the intestinal hygiene of pre-colonoscopy patients:* the indicator was the comparison 2007 vs. 2006, the standard was given by evidence of the form, the weight attributed was 4%, detection was carried out by the Medical Direction and the frequency of acquisition was annual.

3. *To improve professional quality by maintaining a clinical performance indicator system, i.e. the number of outpatient procedures cancelled*. The cancellation of a procedure on the day it was scheduled creates difficulty both for the patient and for the doctor who was scheduled to carry it out [2]. The indicator was the number of procedures cancelled out of the total number of procedures, the standard was 0% (defined according to the IQIP project), the weight was 4%, the means of detection was a verification carried out by the Quality Assurance Office, the data were presented in the hospital's on-line report, and the frequency of acquisition was continual [2].

4. *To continue Colon Cancer Screening Programme*: the indicator was given from the results of the anamnestic questionnaire; the standard was given by evidence of continuation of the programme, the weight was 5% (agreed in the 2007 budget), the means of detection was a verification carried out by the Medical Direction, and the frequency of acquisition was annual.

5. *To improve PEG (percutaneous gastrostomy endoscopy) patient management, reducing the frequency of long-term complications:* the indicator chosen was an increased number of in- and outpatient "endoscopic consultations" with respect to 2006, the standard was >5% (agreed in the 2007 budget), the weight was 9%, the means of detection was a verification carried out by the Digestive Endoscopy Unit, and the frequency of acquisition was annual.

6. *To revise chapters 1, 3 and 6 of the Accreditation Manual: to complete the chapters and accreditation and to pass the internal inspection* [3]. The accreditation documents constituted the indicator, the standard was evidence of the documents, the weight was 10%, the means of detection was linked to the internal inspection, a verification carried out by the Quality Assurance Office, and the frequency of acquisition was continual.

7. *To maintain levels of communication – to provide information about the OU upon patient admission:* the indicator was the number of patients provided with information about the OU upon admission out of the total number of admissions, the standard was 90% (defined by the MAC), the weight was 3%, the means of detection was a verification carried out by the MAC, and the frequency of acquisition was annual.

8. *To keep a "Register of Complications Following Endoscopy":* the indicator chosen was the internal register of the Centre of Digestive Endoscopy, the standard was evidence of the documents (internal register), the weight was 7%, the means of detection was a verification carried out by the Centre of Digestive Endoscopy, and the frequency of acquisition was continual.

9. *Adherence to Unified Appointments Centre (UAC) waiting times (for gastroscopy only):* the waiting times for non-urgent gastroscopy were not to exceed 60 days. The indicator chosen was given by the number of non-urgent gastroscopies with waiting times of < 60 days out of the total number of non-urgent gastroscopies; the standard was 80%, the weight was 10%, the means of detection was internal verification by the Appointments Centre, and the frequency of acquisition of the data was annual [4].

10. *Reporting sentinel events:* "sentinel" in this case is used to describe a particularly serious, avoidable adverse event which could lead to the death of or severe harm to a patient and which causes a loss of faith in the health service in the eyes of the public. One case is sufficient for the organisation to initiate an investigation to identify the factors which could be reduced or eliminated and for adequate corrective measures to be put in place [5]. The indicator chosen was given by specific reports by the OU Managers on actions carried out, the standard was evidence of the reports, the weight was 6%, the means of detection was a verification carried out by the Medical Direction, and the frequency of acquisition was continual.

11. *Quality of procedure:* the indicator was the percentage of times the caecum was reached; the literature indicates a percentage of 90% as an indicator of good colonscopy quality [6]. Cases in which reaching the caecum was not possible or unnecessary were excluded from the calculation, for example right hemicolectomia or insuperable stenosis patients. The standard was >90%, the weight was 13%, the means of detection was a verification carried out by the Centre of Endoscopy, and the frequency of acquisition was annual [6].

12. *Investigation of unforeseen hospitalisations following endoscopic exam:* the indicator was the number of unforeseen patient hospitalisations following endoscopy out of the number of patients who had undergone endoscopy. This indicator permitted identification of the situations which lead to unforeseen hospitalisations in endoscopy

outpatients, and these data may permit us to ascertain of the reasons for inappropriate use of outpatient services. To be included in the calculations, the hospitalisations had to occur within 48 hours of the endoscopic exam and to be unplanned at the time of admission as an outpatient [7]. The standard was <0.45% (mean Project - Wide IQIP), the weight was 9%, the means of detection was a verification carried out by the Medical Direction and the frequency of acquisition was annual.

13. *Risk management: endoscopic decontamination:* the indicator was the number of microbial colonies developing after decontamination. Microbiological monitoring of the endoscopes is an indirect indicator of the adequacy and completeness of the washing/disinfection process and of the application of the recommended protocol, as well as the structural integrity of the instrument. This monitoring may be carried out at different times in the process (after washing/disinfection, after a period of storage). Microbiological monitoring of the endoscopes is a quality check and may thus find application in supplementing verification of criticalities in the implementation of several procedural activities, but should not substitute either diligent supervision of the internal procedures of the entire system or critical management of the infective risk when using endoscopes. Microbiological samples were taken from the flexible endoscopes on a rotary basis and the endoscope-washers were made available in the implementation phase of new procedures. Microbiological sampling of all equipment and endoscope-washers was carried out at least twice a year [7]. The standard was 0% colony development [8]. The weight was 4%, the means of detection was a verification carried out by the Environmental Protection Agency, Ferrara Section, and the frequency of acquisition was six-monthly.

14. *Optimal efficiency levels:* the indicator of this objective was the number of procedures carried out by each operator per year. The standard was 100 gastroscopies per year and 100 colonoscopies per year (this target was decided together with the health workers involved), the weight was 6%, the means of detection was a verification carried out by the Centre of Endoscopy, and the frequency of acquisition was annual.

REFERENCES

1. **Ministero della salute, allegato 9 Raccomandazioni per la pianificazione e l'esecuzione degli screening di popolazione per la prevenzione del cancro della mammella, del cancro della cervice uterina e del cancro del colon retto.** http://www.osservatorionazionalescreening.it/ons/documentazione/raccomandazioni/screening_vers_completa.pdf
2. **International Quality Indicator Project**, http://www.internationalqip.com/index-it-IT.aspx

3. **Norme in materia di autorizzazione e accreditamento delle strutture sanitarie pubbliche e private in attuazione del D.P.R. 14 gennaio 1997, nonché di funzionamento di strutture pubbliche e private che svolgono attività socio - sanitarie e socio – assistenziale**. Regione Emilia Romagna, Legge regionale 12 ottobre1998, n°34,
4. **Intesa tra il Governo, le Regioni e le Province Autonome di Trento e di Bolzano sul Piano Nazionale di contenimento dei tempi di attesa per il triennio 2006-2008,** Conferenza permanente per i rapporti tra lo Stato, le Regioni e le Province autonome di Trento e Bolzano, di cui all'articolo 1, comma 280 della legge 23 dicembre 2005, n.266.
5. **Ministero della Salute, Osservatorio Nazionale sugli eventi sentinella, Protocollo per il monitoraggio degli eventi sentinella, marzo 2008,** http://www.ministerosalute.it/imgs/C_17_pagineAree_238_listaFile_itemName_0_file.pdf
6. **Rex DK, Bond JH, Winawer S *et al*. Quality in tecnical performance of colonscopy and continous quality improvement process for colonscopy.** *Am J Gastroenterol* 2002; 97(6): 1296-308.
7. **Agenzia sanitaria regionale Emilia Romagna, Reprocessing degli endoscopi, indicazioni operative Allegato 4 Protocollo dei controlli microbiologici sugli endoscopi,** Dossier 133; 2006
8. **Douglas B. Nelson, MD; William R. Jarvis, MD et al. Multi-society guideline for reprocessing flexible, Infection control and hospital epidemiology july 2003** http://www.shea-online.org/Assets/files/position_papers/SHEA_endoscopes.pdf
