# Supplementary material for: Multidimensional evaluation of performance: experimental application of the balanced scorecard in Ferrara university hospital
Source: Cost Eff Resour Alloc. 2009 Sep 8;7:15. doi: 10.1186/1478-7547-7-15 (PMC2759901; doi:10.1186/1478-7547-7-15)
Supplement: Additional file 4 — Digestive endoscopy OU - financial resource perspective. the file represents all indicators researched and results obtaines regarding objectives of financial resource perspective of digestive endoscopy. [file 1478-7547-7-15-S4.pdf]

Macro-Objective	Specific Objective	Indicator	Observed value	Standard	Weight	Pictorial representation
Ensure financial sustainability	Increase production levels of 2006	Check Production Report	364,664 **-10.91%** containment of demand; problems with staff absences during the year	409,321	32	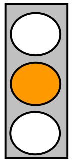
	Check cost report for expenditure/revenue	Report available on line	2006: 45.51% 2007: 57.08%	2007 value =< 2006 value	28	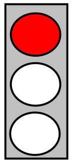
	Check Budget adherence for: 1) Pharmacy 2) Bursary and 3) investments	1) quarterly reports and comparison of estimated and final data; 2) on-line departmental data; 3) quarterly reports and comparison of estimated and final data	Pharmacy - Bursary: 333,027 +3.01% Investments: 56,298.51 -87.58%	2007 value = 2006 value Pharmacy - Bursary: 323,286 investments: 453,440	40	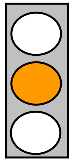

FINANCIAL RESOURCE PERSPECTIVE

The specific objectives identified were:

1. *To increase 2006 production levels:* the indicator was the on-line Control Report of the hospital; the chosen target was the value produced in euro in 2007, established in the budget. The weight was 32%, the means of detection was a verification carried out by Management Control, and the frequency of acquisition was six-monthly.

2. *To check “Expenditure/Revenue Report”:* the indicator was the Report available on line, and the target chosen was the value acquired in 2007, which was to be inferior or equal to the value acquired in 2006 (defined in the 2007 budget). The weight was 28%, the means of detection was a verification carried out by Management Control, and the frequency of acquisition was six-monthly.
3. *Budget adherence/check (for Pharmacy, Bursary, and investments):* the indicator was given by quarterly on-line Reports and was based on comparison of the preventative budget and actual spending, and the on-line budget forms of the department. The standard chosen was given by the 2007 Bursary values equal to the 2006 values plus € 30,000 (agreed in the 2007 budget), the weight was 40%, the means of detection was a verification carried out by Management Control, and the frequency of acquisition was six-monthly.
