# Supplementary material for: Multidimensional evaluation of performance: experimental application of the balanced scorecard in Ferrara university hospital
Source: Cost Eff Resour Alloc. 2009 Sep 8;7:15. doi: 10.1186/1478-7547-7-15 (PMC2759901; doi:10.1186/1478-7547-7-15)
Supplement: Additional file 5 — Digestive endoscopy OU - growth and learning perspective. the file represents all indicators researched and results obtaines regarding objectives of growth and learning perspective of digestive endoscopy. [file 1478-7547-7-15-S5.pdf]

Macro-Objective	Specific Objective	Indicator	Observed value	Standard	Weight	Pictorial representation
**HUMAN ASSETS**	Refresher courses for all staff	Number of credits/year/employee	30	>=30	40	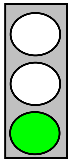
	Improve interpersonal skills	Number of complaints about interpersonal skills	0	0	20	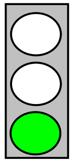
**ORGANISATIONAL ASSETS**	Satisfaction of operator: Organisational wellbeing	Internal questionnaire	2004: 5.21<br>2001: 4.52	Detection of mean value of organisational wellbeing > past (2004 value > 2001 value)	40	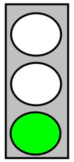

GROWTH AND LEARNING PERSPECTIVE

The specific objectives identified were:

1. *Refresher courses for all staff members:* the indicator was the number of Continuing Medical Education (CME) credits per year per staff member. Significance: the professional capacity of a health worker can be divided into three main characteristics: possession of up-to-date technical knowledge (knowing), possession of

technical or manual skills (doing), and possession of communicative and interpersonal skills (being). The rapid and continuous development of medicine and, in general, biomedical knowledge, as well as the continual progress in technological and organisational innovations, make it difficult for a health worker to maintain these three characteristics at the maximum level. To this end, the worldwide Continuing Medical Education programme was conceived; this consists of the organisation and control of all practical and theoretical training activities promoted by various organisations (scientific societies, professional organisations, hospitals or establishments specialised in health training, etc.), with the aim of keeping health workers' professional capacity at a high level and up to date. CME is aimed at evaluating the training events, so that each physician, nurse, or other health worker may be guaranteed quality and useful training to safeguard their professionalism; in our country, CME is governed by specific laws: legislative decree 502, 30th December 1992, completed by legislative decree 229, 19th June 1999 [1, 2]. Elaboration of the CME programme was entrusted, according to art. 16.3 of the aforementioned legislative decree, to the National Commission for Continuing Education, which has the task of defining the number of credits to be acquired by an operator in a certain time period and of defining the requisites for accreditation of public and private entities which carry out these training activities. CME credits are a measure of the commitment and time each health worker has dedicated per year to improving the quality of their professionality [3]. The standard was >=30, the weight was 40%, the means of detection was a verification carried out by the Training and Updating staff, and the frequency of acquisition was annual [2].

2. *Satisfaction of the operators or organisational wellbeing :* the indicator was an internal questionnaire, the standard was the detection of a mean value of improvement in organisational wellbeing with respect to the past, the weight was 40%, the means of detection was a verification carried out by the Quality Assurance Office, and the frequency of acquisition was annual.

3. *To improve interpersonal skills:* the indicator chosen was the number of complaints regarding treatment programmes, staff availability and helpfulness, and provision of information to the health service users and their family members. The standard was 0 complaints of this type, the weight was 20%, the means of detection was a verification carried out by the Public Relations Office, and the frequency of acquisition was annual.

REFERENCES

1. **Presidenza della Repubblica, decreto legislativo 19 giugno 1999, n. 229 “Norme per la razionalizzazione del Servizio Sanitario Nazionale,** pubblicato nella *Gazzetta Ufficiale* n. 165 del 16 luglio 1999 - Supplemento Ordinario n. 132
2. **Ministro della Salute, Circolare 5 marzo 2002 con oggetto: ECM - Formazione continua** http://www.ministerosalute.it/ecmimgs/C_18_normativa_10_listafile_file_0_linkfile.pdf
3. **Ministero della Salute, Il Programma nazionale di educazione continua in medicina** http://www.ministerosalute.it/ecm/presentazione/presentazione.jsp?sez=pro
