# Supplementary material for: Multidimensional evaluation of performance: experimental application of the balanced scorecard in Ferrara university hospital
Source: Cost Eff Resour Alloc. 2009 Sep 8;7:15. doi: 10.1186/1478-7547-7-15 (PMC2759901; doi:10.1186/1478-7547-7-15)
Supplement: Additional file 6 — Strategic map of analysis laboratory OU. the file represents the final outcome of strategic map of analysis laboratory. [file 1478-7547-7-15-S6.pdf]

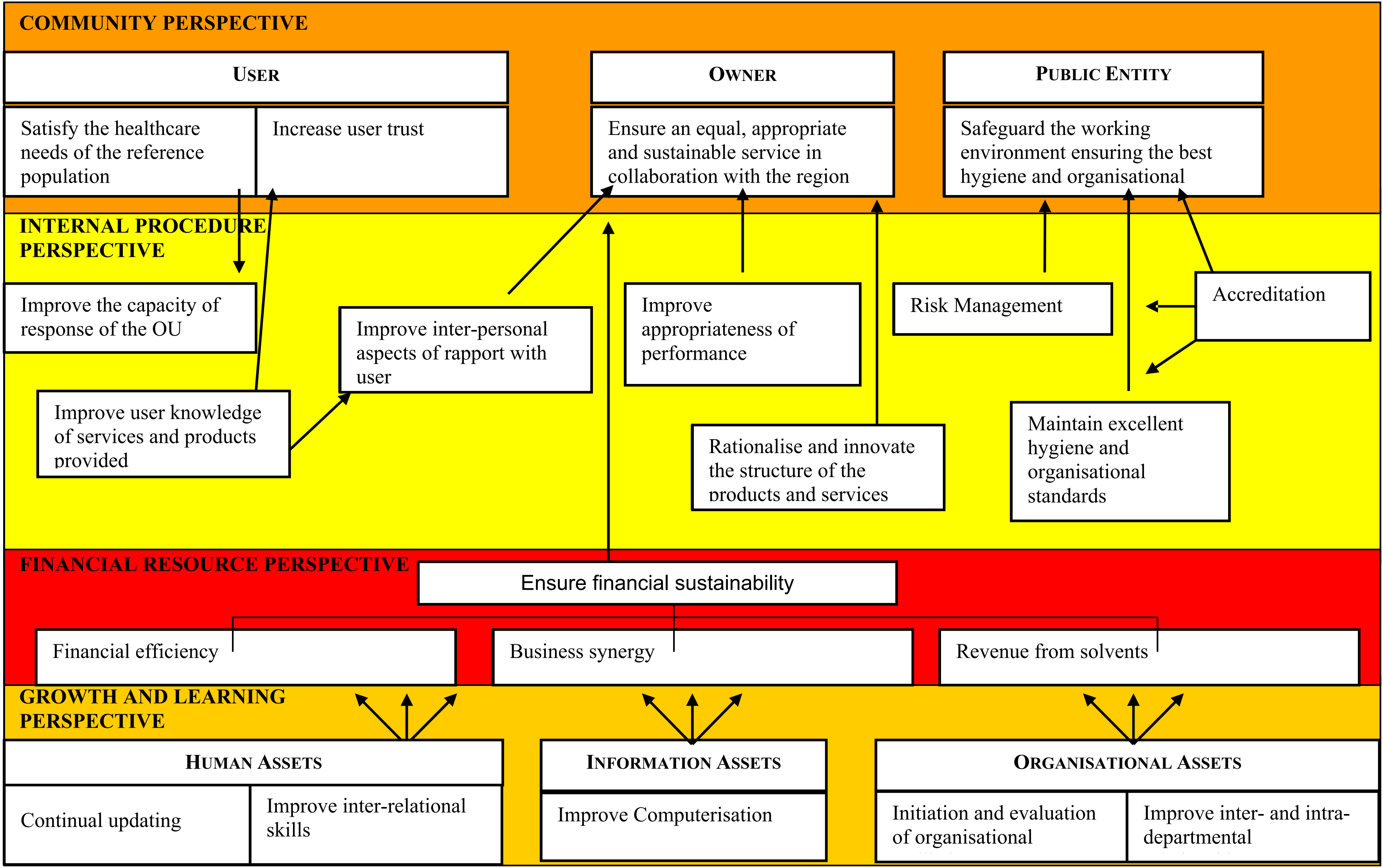
COMMUNITY PERSPECTIVE
USER
Satisfy the healthcare needs of the reference population
Increase user trust
OWNER
Ensure an equal, appropriate and sustainable service in collaboration with the region
PUBLIC ENTITY
Safeguard the working environment ensuring the best hygiene and organisational
INTERNAL PROCEDURE PERSPECTIVE
Improve the capacity of response of the OU
Improve inter-personal aspects of rapport with user
Improve appropriateness of performance
Risk Management
Accreditation
Improve user knowledge of services and products provided
Rationalise and innovate the structure of the products and services
Maintain excellent hygiene and organisational standards
FINANCIAL RESOURCE PERSPECTIVE
Ensure financial sustainability
Financial efficiency
Business synergy
Revenue from solvents
GROWTH AND LEARNING PERSPECTIVE
HUMAN ASSETS
Continual updating
Improve inter-relational skills
INFORMATION ASSETS
Improve Computerisation
ORGANISATIONAL ASSETS
Initiation and evaluation of organisational
Improve inter- and intra-departmental
