# Supplementary material for: Multidimensional evaluation of performance: experimental application of the balanced scorecard in Ferrara university hospital
Source: Cost Eff Resour Alloc. 2009 Sep 8;7:15. doi: 10.1186/1478-7547-7-15 (PMC2759901; doi:10.1186/1478-7547-7-15)
Supplement: Additional file 7 — Analysis laboratory OU - community perspective. the file represents all indicators researched and results obtaines regarding objectives of community perspective of analysis laboratory. [file 1478-7547-7-15-S7.pdf]

Macro.Objective	Specific Objective	Indicator	Observed value	Standard	Weight	Pictorial representation
**USER**<br><br>Satisfy the healthcare needs of the reference population	Increase user trust	N° of complaints	2007: 3 complaints	< 2 complaints/year	20	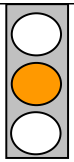
	Timeliness	Internal turn-around time (TAT):<br>1) Emergencies: TAT <30 min./total emergency requests<br>2) Urgencies: TAT <2 h/total urgent requests	1) 8.1%<br>2) 38.1%<br>(detection period: 1/07/08-22/09/08)	1) 70%<br>2) 90%	12	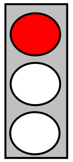
		External turn-around time (TAT):<br>1) TAO internet: TAT by 12:30 p.m. of the following day/total OACT internet request<br>2) SOLE: TAT within 1 day of request completion/total requests	1) 97.3%<br>2) 100%<br>(first six months of 2008)	>95%	12	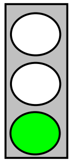
	Convenience	Various means of report reception: (direct delivery, by mail, by internet)<br>1) Number of reports delivered by post/total reports<br>2) Num. Reports number of reports delivered by SOLE (where activated)<br>3) Number of reports delivered by TAO INTERNET (in activated districts)	1) 1.86%<br>2) 3,462<br>3) 1,658<br>data from four-month period in 2008	1) >5%<br>2) >3000 (four-month period)<br>3) >1000 (four-month period)	10	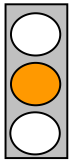
		Automated withdrawal points in Central Health Structures	100% (2008)	100%	5	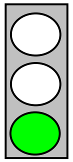

**OWNER**<br><br>Ensure an equal, appropriate and sustainable service in collaboration with the region	Budget result	Final balance in line with estimated budget	Difference: 5.4% (2007)	alignment	6	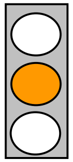
**PUBLIC ENTITY**<br><br>Safeguard the working environment ensuring the best hygiene and organisational	Ease of access	Wait for access/working days	appointments: 5.4 facilitated categories: acceptable (Jan-Jul 2008)	appointments:<5 working days facilitated categories: free access without appointment >10% for 90% of working days	20	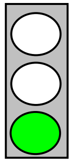
		Appointment Points	100% (2008)	UAC list and Pharmacy with UAC function: 100%	5	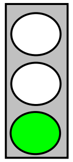
		Consulting rooms open/total consulting rooms (work days, Saturday)	100% (first six months of 2008)	Work days: 100% Saturday: at least 1 consulting room per district	5	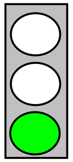
	Transparency	Employees in contact with public who wear ID badges/total employees	100% (2007)	95% of employees	5	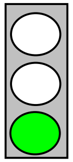

COMMUNITY PERSPECTIVE

The specific objectives of this perspective were:

1. *Budget result:* The indicator was given by comparison of the final (observed) value with the estimated (forecast) value, and the standard was alignment (defined in the 2007 budget), the weight was 6%, the means of detection was a verification carried out by Management Control and the frequency of acquisition was annual.

2. *To increase user trust:* the indicator was the number of complaints and recommendations received, the standard was < 2/year (defined with the health workers based on past experience: one case in 2005, and two cases in 2006), the weight was 20%, the means of detection was a verification carried out by the PRO and the frequency of acquisition was continual.

3. *Ease of access:* for this objective,three indicators were identified:

   – *Waiting times for access to the service/working days*: for this indicator two targets were selected (defined with the health workers based on previous years' experience):

     o Number of patients with appointments <5 working days for 80% of working days;

     o Facilitated capacities: free access without appointment >10% for 90% of working days.

   The weight was 20%, and the means of detection was a verification carried out by the *Public Healt Unit* of our Province and the frequency of acquisition was quarterly.

   – *User Appointment Points/Total appointment points (Unified Appointments Centre):* the standard (defined in agreement with the health workers on the basis of past experience) was 100%, the weight was 5%, and the means of detection was a verification carried out by the *Public Healt Unit* of our Province, and the frequency of acquisition was annual.

- *Consulting rooms open/total consulting rooms (work days and Saturdays)*: the standard was 100% per work day; and at least one laboratory open per Saturday (defined in agreement with the health workers on the basis of past experience), the weight was 5%, the means of detection was a verification carried out by the *Public Healt Unit* of our Province, and the frequency of acquisition was annual.

4. *Convenience:* for this objective two indicators were identified:

   - *Various types of report reception:* (direct delivery, by mail, by internet): a) Number of reports delivered by mail/total reports, b) number of reports delivered by the on-line health project SOLE (where activated), c) Number of reports delivered by internet (in activated districts). The SOLE project is a non-compulsory information network which connects the 3,800 or so GPs and paediatricians with all health facilities and specialist physicians in the Emilia Romagna region Health Authority. SOLE facilitates the handling of the 4 million plus citizens in the region via the sharing of health information between the physicians treating each patient. Requests for tests and examinations, medical reports, and hospital discharge data are all available on the SOLE network, an advantage for both the physicians and their patients [1]. The availability of reports at the doctor's surgery (and, where applicable, to the public via internet or mail) saves a significant amount of time and movement and therefore increases timeliness in diagnosis and commencement of treatment by the GP. The reports of patients subject to oral anticoagulant therapy can also be sent via the electronic information network: this guarantees greater timeliness and more convenience for the patients. The standard (defined in agreement with the health workers on the basis of previous years' experience): was, respectively: a) >5%, b) >3000, and c) >1000. The weight was 10%, the means of detection was a verification carried out by the Analysis Laboratory, and the frequency of acquisition was six-monthly.

   - *Automated withdrawal points in the central health facilities:* the standard was 100%, the weight was 5%, the means of detection was a verification carried out by the *Public Healt Unit* of our Province, and the frequency of acquisition was annual.

5. *Timeliness:* two indicators were identified:

   - *Internal turn-around time* (TAT): 1) Emergencies: TAT <30 minutes/total emergency requests; 2) Urgencies: TAT <2 hours/total urgent requests. The *turn-around time* of the laboratory tests was defined as the time taken from the clinician's request for a patient's biological data to the clinician's receipt of said data. This time was the result of the sum of the TATs of the numerous pre-analytical, analytical and post-analytical steps which make up the entire process

[2]. The TAT can be considered an indicator of overall organisational quality and describes the capacity of a health facility to acquire and utilise (or to supply and make available) information necessary for diagnostic and therapeutic ends [3]. Reduction in TAT is an objective of the entire hospital, not only the Laboratory [4]. The standard: was (defined in agreement with the health workers on the basis of past experience) respectively: 1) 70%, and 2) 90%, the weight was 12%, the means of detection was a verification carried out by the Analysis Laboratory, and the frequency of acquisition was annual.

- *External turn-around time* (TAT): 1) via internet for the patients undergoing oral anti-coagulant treatment (OACT): TAT before 12,30 p.m. of the following day/total OACT internet request; 2) for project SOLE: TAT response completion within 1 day/total requests. The standard was >95%, the weight was 12%, the means of detection was a verification carried out by the Analysis Laboratory and the frequency of acquisition was annual.

6. *Transparency:* the indicator was the percentage of employees dealing with the public who wear ID badges, the standard was 95% of employees (defined by the MAC, PRO and the Quality Assurance Office), the weight was 5%, the means of detection was a verification carried out by the MAC, and the frequency of acquisition was annual.

REFERENCES

1. **Sito web italiano del progetto SOLE (sanità on line):** http://www.progetto-sole.it/consultazione/home.php
2. **Hawkins RC Laboratory turnaround time**. Clin Biochem Rev 2007 , 28(4): 179-194
3. **LabNews, anno 9, numero 35, giugno 2007** http://www.qualitologia.it/formazione/17.pdf
4. **ESADIA, anno 3, num 5, luglio 2000,** http://www.roche-diagnostics.it/pubblicazioni/esadia/2000/ESA5.pdf
