# Supplementary material for: Multidimensional evaluation of performance: experimental application of the balanced scorecard in Ferrara university hospital
Source: Cost Eff Resour Alloc. 2009 Sep 8;7:15. doi: 10.1186/1478-7547-7-15 (PMC2759901; doi:10.1186/1478-7547-7-15)
Supplement: Additional file 8 — Analysis laboratory OU internal procedure perspective. the file represents all indicators researched and results obtaines regarding objectives of internal procedure perspective of analysis laboratory. [file 1478-7547-7-15-S8.pdf]

Macro-Objective	Specific Objective	Indicator	Observed value	Standard	Weight	Pictorial representation
Risk Management	Sentinel Events: reporting Sentinel Events	Specific reports of OU Manager on actions carried out	The specific reports have been produced	evidence reports	5	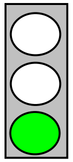
Improve inter-personal aspects of rapport with user	Maintain levels of communication	Verification by MAC of information sheet provision upon admission	MAC report has been produced	Report of MAC	5	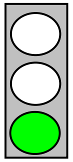
Accreditation	Prepare accreditation	Internal inspection	The specific documents have been produced	Evidence: relevant documents	10	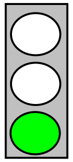

Rationalise and innovate the structure of the products and services<br><br>Improve user knowledge of services and products provided<br><br>Improve the capacity of response of the OU	Constitution of integrated H&S network	Number and type of integrations and agreements with other health agencies/year	12	>6	10	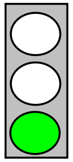
	Improvement of appointments service	Number of GPs in the network (SOLE-OACT INTERNET)/GPs in central-northern district of Ferrara	46.4% (2008)	>30%	5	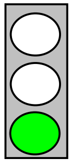
		Appointment reports/free access: samples in free access/total samples	19% (first six months of 2008)	>10%	5	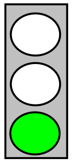
	Optimisation of acceptance and sampling procedure	Mean samples per operator/premises/hour	23/hour (first six months of 2008)	>22/hour	12	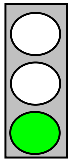
	Optimisation of Analysis and Validation Report/quality procedure	Responsibility grid: functional units applying grid/total functional units (Accreditation)	100% (2007)	100%	3	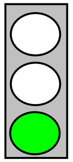

		Intra-lab reproducibility of results (CV%)	all the values are in the range (first six months of 2008)	<5% for 80% of chemical/clinical tests; <10% for 50% of immunometric tests	8	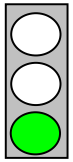
		Inter-Lab comparability of reports (EQAS-Emilia Romagna Region): analytes participating in EQAS/total analytes	100% (first six months of 2008)	>90%	8	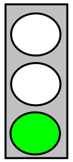
Improve appropriateness of performance	Chemical and clinical analytical accuracy	MISA (indicator of performance: global evaluation of Laboratory)	2007: 81	>= 101 - 150 MAD (acceptable performance) 50 - 100 (good performance)	15	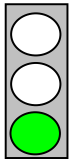
	Optimisation of urgent analyses procedure	Turn-around time (TAT) Urgencies: TAT <2 h /total urgent requests	38.1% (3564/5787) (detection period: 1/07/08-22/09/08)	90%	14	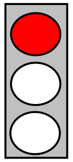

Internal procedure Perspective

The specific objectives of this perspective were:

1. *Reporting sentinel events:* the indicator chosen was given by specific reports by the OU Managers on actions carried out, the standard was given by evidence of the actions (defined with the health workers in the 2007 budget), the weight was 5%, the means of detection was a verification carried out by the Medical Direction, and the frequency of acquisition was continual [1].

2. *To maintain levels of communication:* the indicator was verification by the MAC regarding the provision of information about the OU upon acceptance, the standard was evidence of the delivery of information, the weight was 5%, the means of detection was a verification carried out by the MAC, and the frequency of acquisition was annual.

3. *To prepare for Accreditation:* the indicator was an internal inspection, the standard was the positive judgement of the internal inspection, the weight was 10%, the means of detection was a verification carried out by the Quality Assurance Office, and the frequency of acquisition was continual [2].

4. *Constitution of Hub & Spoke integrated network:* the indicator was the number and type of agreements and integrations with other health agencies per year. The financial law of 2007, article 1 point 96, required reorganisation of the public structure laboratory diagnostic network. Consequently the Ministry of Health and the Ministry of Economy and Finance arranged for this to happen (issuing guidelines on the contents of the laboratory network plan) [3]. Due to the increased pressure to change which characterised laboratory medicine, various laboratory network reorganisation models were developed in Italy, with consequent spiralling increases in costs. Thus the Ministry of Health and the Ministry of Economy and Finance issued indications for constructing a homogeneous national system to develop integrated networks of laboratory points in the regional health authorities. In this context the Emilia Romagna region had already developed, in the Regional Health Plan of 1999–2001, a Hub & Spoke organisational model of services. This model involves the concentration of health care production of greater complexity in centres of excellence (Hubs) and the functional arrangement of peripheral centres under them (Spokes), which mainly deal with the selection and dispatch to the hub centres [4]. The standard was >6 (defined with the health workers on the basis of previous years' experience), the weight was 10%, the means of detection was a verification carried out by the Analysis Laboratory, and the frequency of acquisition was annual.

5. *Improvement of appointment services:* the following indicators were chosen: 1) number of GPs in the network (SOLE-AOCT INTERNET)/number of GPs in the central-northern district of Ferrara; 2) appointment report/free access: number of samples taken in free access/total number of samples taken. The standard (defined with the health workers on the basis of previous years' experience) was, respectively: 1) >30% and 2) >10%, the weight was, respectively: 1) 5% and 2) 5%, the means of detection was a verification carried out by the Analysis Laboratory, and the frequency of acquisition was annual.

6. *Optimisation of acceptance and sampling processes:* the indicator was the mean samples taken per operator/premises/hour. This was significant for enabling the health workers to optimise their work. The standard (defined with the health workers on the basis of previous years' experience) was >22/hour, the weight was 16%, the means of detection was a verification carried out by the Analysis Laboratory, and the frequency of acquisition was annual.

7. *Optimisation of the Analysis and Validation Report/quality procedure:* the following indicators were chosen: 1) responsibility grid: functional units applying the grid/total functional units (accreditation); ISO EN 17025 suggests the construction of a requirement/responsibility grid which attributes responsibilities in the fulfilment of each requirement to every member of the group and 2) intra-laboratory reproducibility of results (CV%) [5]. The coefficient of variation (CV%), or the relevant standard deviation, was the measure of the relative imprecision calculated as a relationship between standard deviation and mean multiplied by 100. The precision parameter was linked to the concept of analytical reproducibility. Precision indicated agreement between measurements carried out on the same sample (intra-assay) or analogous samples (inter-assay) obtained via analyses carried out on the same day (intra-day) or on different days (inter-day). This parameter was evaluated via determination of the standard deviation and coefficient of variation (CV), a percentual indicator which correlated errors in measurement to the average value of concentration obtained during repeated analysis of the same sample. These parameters should be measured using a minimum of five determinations per concentration. A minimum of three levels of concentration within the range of expected concentration values are recommended. Coefficients of variation and standard deviations supplied the degree of precision of the analyses, i.e. an idea of the reproducibility of the analyses. 3) inter-laboratory comparability of reports (EQAS Emilia Romagna Region): analytes participating in EQAS programmes/total analytes. The Quality Assurance and External Verification of the Quality of Health Care and Medical Treatment (QA and EQAS) bodies utilise a methodology which subjects the evaluation, by the governing bodies, of the quality of health care, with a view to improving it where necessary. The EQAS methodology is based on the concept that, to evaluate the quality of a Health Care Provider, it is necessary to develop criteria and standards which can be compared with the levels of health care actually being provided. The external quality evaluation programme to which the Analysis Laboratory OU of Ferrara University Hospital adheres to was the first EQAS service organised by a public entity in Italy. Promoted by the Emilia Romagna Region in

1987, the programme was organised and managed by Bologna S.Orsola Malpighi University Hospital [6]. EQAS is a system of continual monitoring of laboratory performance in order to evaluate the reliability of the results for each analyte and to compare the results of all participant laboratories. The objective of the programme was improvement in quality of performance of the laboratories via the production and circulation of data. This allows the laboratories to compare the reliability of their performance with that of other laboratories, facilitating identification of the areas in which the determinations are still unreliable and stimulating improvement in the reliability of the tests carried out. The standard (defined with the health workers on the basis of past experience and indications from the quality programmes) were: 1) 100%, 2) <5% for 80% chemical clinical tests; <10% for 50% immunometric tests, and 3) >90%, respectively, the weights were: 1) 5%, 2) 5%, and 3) 5%, respectively, the means of detection was a verification carried out by the Analysis Laboratory, and the frequency of acquisition was continual.

8. *Clinical and chemical analytical accuracy:* the indicator was MISA (indicator of performance: global evaluation of the laboratory). The accuracy was an indicator of the vicinity of the result obtained to the real value (theoretical). Accuracy was determined by analysing samples of known analytical quantities; the value of the concentration obtained from the analysis was then compared with the real quantity (nominal), and the percentage difference was evaluated. The Analysis Laboratory participates in the EQAS programmes organised by the Castelfranco Veneto Centre for Biomedical Research. The Castelfranco Veneto Centre for Biomedical Research has long interpreted external evaluation of quality as a tool for total quality management. In this context the EQAS programmes aim for continual improvement in analytical performance in the lab, and stimulate the workers to review not only the analytical procedures, but also each step in the process, from sample collection to communication of information. The scheme requires communication of results via a report in use in the laboratory, with the relevant reference intervals. The results are then reviewed so that a judgement can be made for each analytical performance, and thus a global evaluation of the laboratory which also takes into account non-analytical errors. The objectives of quality for each analyte were defined in terms of difference (D) from the value assigned (median of the results per homogenous method group), compared with the assigned coefficient of variation which was obtained from the State of the Art. Based on the absolute value of the difference (AD), the performance was classified as excellent (<50), good (50-100), sufficient (101-150) or insufficient (>150). The overall performance of the laboratory was evaluated in terms of MISA i.e. as a mean of all the values of AD>150, to which the insufficient performance values (PNA), the outliers (OUT) and the errors found in the report were added [7]. The standard was from >= 101 to 150 MISA (sufficient), the weight was 15%, the means of detection was a verification carried out by the Quality Assurance Office, and the frequency of acquisition was annual.

9. *Optimisation of urgent analyses procedure:* the indicator was the turn-around time (TAT) for urgencies: TAT <2 hours/total urgent requests, the standard was 90%, the weight was 14%, the means of detection was a verification carried out by the Analysis Laboratory, and the frequency of acquisition was six-monthly.

REFERENCES

1. **Ministero della Salute, Osservatorio Nazionale sugli eventi sentinella, Protocollo per il monitoraggio degli eventi sentinella, marzo 2008,** http://www.ministerosalute.it/imgs/C_17_pagineAree_238_listaFile_itemName_0_file.pdf
2. **Agenzia Sanitaria Regionale Emilia Romagna, Requisiti specifici per l'accreditamento delle strutture di Patologia Clinica (Laboratorio Analisi)** http://www.regione.emilia-romagna.it/agenziasan/aree/accred/accreditamento/requisiti_spec/patologia_clinica.pdf
3. **Ministero della Salute e Ministero dell'Economia, legge n. 296/2006 (finanziaria 2007), art. 1, comma 796, lettere o, s, t, u** http://www.camera.it/parlam/leggi/06296l.htm
4. **Regione Emilia Romagna, Piano sanitario regionale 1999-2001** http://www.regione.emilia-romagna.it/sanita/psr/piano.htm
5. **International Standards for Business, Government and Society,** Norma ISO EN 17025 – 2005 General requirements for the competence of testing and calibration laboratories
6. **Azienda Ospedaliero-Universitaria di Bologna Policlinico S.Orsola Malpighi, Programmi di valutazione esterna di Qualità** http://www.aosp.bo.it/veq/homepage.htm
7. **Secchiero S., Zardo L., Sciacovelli L., Plebani M. Centro di Ricerca Biomedica, Castelfranco Veneto (TV) – VEQ per biochimica clinica: stima della variabilità analitica interlaboratori e degli errori di refertazione** http://www.centroricercabiomedica.it/doc/Pubblicazioni/Poster2004%20Urine.pdf
