# Supplementary material for: Multidimensional evaluation of performance: experimental application of the balanced scorecard in Ferrara university hospital
Source: Cost Eff Resour Alloc. 2009 Sep 8;7:15. doi: 10.1186/1478-7547-7-15 (PMC2759901; doi:10.1186/1478-7547-7-15)
Supplement: Additional file 9 — Analysis laboratory OU -financial resource perspective. the file represents all indicators researched and results obtaines regarding objectives of financial resource perspective of analysis laboratory. [file 1478-7547-7-15-S9.pdf]

**Macro-Objective**	**Specific Objective**	**Indicator**	**Observed value**	**Standard**	**Weight**	**Pictorial representation**
**Ensure financial sustainability**	Maintain Provincial production levels	Report of production	2006: 4,947,668 2007: 5,423,331 The objective was indicative in difficult forecast for 2008	2007 production values for = 2006 production values	20	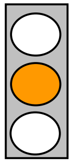
	Cost containment action	Comparison report of production/contribution margin	3,252,614 The objective was indicative in difficult forecast for 2008	2,356,474	25	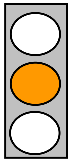
	Check budget adherence: 1) Pharmacy and Bursary 2) investments, 3) external contractors	1) quarterly report comparing real and expected expenditure, 2) on-line departmental information 3) quarterly report comparing real and expected expenditure	1) 4,041,503<br>2) 6,983,082<br>3) 50,932	1) 4,027,088<br>2) 10,594,700 (department)<br>3) 51,282	30	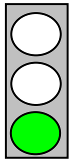
	Check cost report for expenditure/revenue	On-line report	2006:67.64%<br>2007:60.52%	value 2007 =< value 2006	20	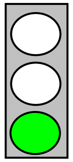
	Business synergy	Assets from ticket taking	1,162,395.6 (first six months 2008) 2008 estimate: 2,324,791.1	>1,800,000 euro/year	5	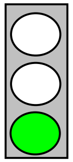

Financial Resources Perspective

The specific objectives of this perspective were:

1. *To maintain Provincial production levels:* the indicator was a report of production values, the standard was given by 2007 production values, which had to be equal to the 2006 production values (defined in the 2007 budget). The weight was 20%, the means of detection was a verification carried out by Management Control, and the frequency of acquisition was quarterly.

2. *Cost containment:* the indicator was the contribution margin, the standard was the value provided for in the 2007 budget, the weight was 25%, the means of detection was a verification carried out by Management Control, and the frequency of acquisition was quarterly.

3. *Check Budget Adherence: for the Pharmacy/Bursary, investments and external contractors:* the indicators were given by quarterly reports comparing actual and estimated balances and by the on-line Department budget. The standard was the respective value in euro provided for in the 2007 budget, the weight was 30%, the means of detection was a verification carried out by Management Control and by the Human Resources Management Direction, and the frequency of acquisition was quarterly, continual, and quarterly, respectively.

4. *Check cost report for expenditure/revenue:* the indicator was the on-line report, the standard was defined in the 2007 budget, the weight attributed was 20%, the means of detection was a verification carried out by Management Control and by the Human Resources Management Direction, and the frequency of acquisition was six-monthly.

5. *Business synergy:* the indicator was the assets from ticket takings, the standard was agreed on the basis of supply contracts, the weight was 5%, the means of detection was a verification carried out by the Analysis Laboratory, and the frequency of acquisition was annual.
