# Supplementary material for: Multidimensional evaluation of performance: experimental application of the balanced scorecard in Ferrara university hospital
Source: Cost Eff Resour Alloc. 2009 Sep 8;7:15. doi: 10.1186/1478-7547-7-15 (PMC2759901; doi:10.1186/1478-7547-7-15)
Supplement: Additional file 10 — Analysis laboratory OU - growth and learning perspective. the file represents all indicators researched and results obtaines regarding objectives of growth and learning perspective of analysis laboratory. [file 1478-7547-7-15-S10.pdf]

Macro-Objective	Specific Objective	Indicator	Observed value	Standard	Weight	Pictorial representation
**Human Assets**<br><br>Continual updating<br><br>Improve inter-relational skills	Refresher courses for all the staff	Number of credits/year/employee	Number of credits 2007/ employee: > 30	>=30	15	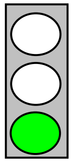
	Improve interpersonal skills	Number of complaints regarding interpersonal skills	0 (2007)	0	10	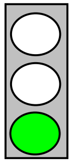
	Teaching and research activity	Participation in research programmes/year	1	=>1	20	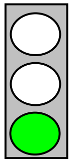
		Teaching and tutoring activity	7 (2007)	OU staff involvement in teaching in at least 10 courses	20	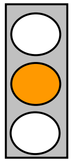
**Organisational Assets**<br><br>Initiation and evaluation of organisational wellbeing<br><br>Improve inter- and intra-departmental relationships	Satisfaction of operators/ Organisational wellbeing	Organisational wellbeing questionnaire	2004: 5.21<br>2001: 4.52	Mean detection value of organisational wellbeing > past (2004 value > 2001 value)	30	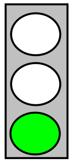

**Information Assets**<br><br>Improve Information Comunication Tecnology	Information Tecnology	Number of GPs connected in network (SOLE/OACT INTERNET)/ GPs in central-northern district of Ferrara	46.4% (2008)	>30%	5	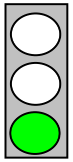

Growth and Learning Perspective

The specific objectives of this perspective were:

1. *Refresher training for all staff members:* the indicator was the number of credits/year/employee, the standard was >=30 (CME), the weight was 15%, the means of detection was a verification carried out by the Analysis Laboratory, and the frequency of acquisition was annual.

2. *Organisational wellbeing:* the indicator was a questionnaire on organisational wellbeing: the detection value was mean organisational wellbeing, the standard was given by the value for the year 2004 > value for the year 2001, the weight was 30%, the means of detection was a verification carried out by the Quality Assurance Office and the frequency of acquisition was annual.

3. *Teaching and research activity:* two indicators were chosen: 1) participation in research programmes/year, 2) teaching and tutoring activities; the standard (defined on the basis of past experience) was 1) >1, and 2) Service staff teaching involvement in at least 10 courses, respectively, and the weight for both was 20%. The means of detection was a verification carried out by the Analysis Laboratory, and the frequency of acquisition was annual.

4. *Information-communication technology (ICT):* the indicator was the number of GPs connected on line. An increase in ICT facilitates the exchange of information between the Analysis Laboratory and the GPs and is an opportunity for staff growth. The standard was >30% of the GPs in the central-northern district of Ferrara

(defined on the basis of past experience), the weight was 5%, the means of detection was a verification carried out by the *Public Healt Unit* of our Province, and the frequency of acquisition was annual.

5. *Improve interpersonal skills:* the indicator was the number of complaints regarding this issue, i.e. complaints regarding the transmission of information to the users and their family members, course of treatment, and availability for consultation. The standard was 0 complaints (defined on the basis of past experience), the weight was 10%, the means of detection was a verification carried out by the PRO, and the frequency of acquisition was annual. The standard “0” has been proposed by UO because it was the performance of the 2006. For this reason the target has been considered acceptable.
