# Supplementary material for: Multidimensional evaluation of performance: experimental application of the balanced scorecard in Ferrara university hospital
Source: Cost Eff Resour Alloc. 2009 Sep 8;7:15. doi: 10.1186/1478-7547-7-15 (PMC2759901; doi:10.1186/1478-7547-7-15)
Supplement: Additional file 11 — Digestive endoscopy OU - BSC. the file represents the global performance reached in four perspectives of strategic map of digestive endoscopy. [file 1478-7547-7-15-S11.pdf]

Perspective	Pictorial representation
COMMUNITY	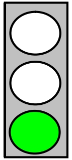
INTERNAL PROCEDURE	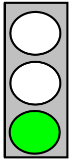
FINANCIAL RESOURCE	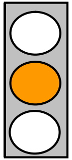
GROWTH AND LEARNING	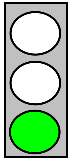
